# Cushing’s Syndrome associated with diabetes mellitus in a Persian cat: clinical features, diagnosis, and therapeutic management

**DOI:** 10.29374/2527-2179.bjvm004026

**Published:** 2026-08-12

**Authors:** Laura Gonçalves Nascimento, Roberta Oliveira de Carvalho, Flaviani Emília dos Santos, Vitor Vieira de Souza, Diego Ribeiro, Maria Luiza Maciel de Mendonça, Fernando Yoiti Kitamura Kawamoto

**Affiliations:** 1 Centro Universitário de Lavras (UNILAVRAS), Lavras, MG, Brazil; 2 Universidade Federal de Minas Gerais (UFMG), Belo Horizonte, MG, Brazil; 3 Faculdade Qualittas, Belo Horizonte, MG, Brazil; 4 Universidade Estadual Paulista (UNESP), Botucatu, SP, Brazil

**Keywords:** hyperadrenocorticism, hypercortisolism, felines, hyperglycemia, hiperadrenocorticismo, hipercortisolismo, felinos, hiperglicemia

## Abstract

The association between Cushing's syndrome (CS) and diabetes mellitus (DM) in cats is rarely reported. Complications related to hypercortisolism and persistent hyperglycemia may occur, which may complicate the control of both endocrinopathies. This report describes the case of a 10-year-old Persian cat diagnosed with CS and DM. The patient presented with polyuria, polydipsia, chronic dry seborrhea on the dorsal region and flanks, and lack of hair regrowth on the abdomen. Through the dexamethasone suppression test (0.1 mg/kg) and the presence of persistent hyperglycemia with elevated fructosamine levels and glycosuria, diagnoses of Cushing's syndrome and DM were established. Treatment was initiated with trilostane (2 mg/kg PO BID) for CS and insulin glargine (2 IU/animal SC BID) for DM, resulting in stabilization of blood glucose, cortisol concentrations, and the patient's clinical condition, although cutaneous fragility persisted for a prolonged period. Diagnostic investigation for hypercortisolism should be considered in cats with DM to allow earlier diagnosis, reduce underdiagnosis, and improve the availability of epidemiological and clinical data on this association. The diagnostic approach and clinical management described may aid veterinarians in recognizing this coexistence and anticipating therapeutic challenges, such as persistent cutaneous fragility despite cortisol control.

## Resumo

A associação entre a síndrome de Cushing (SC) e o diabetes mellitus (DM) em gatos é raramente relatada. Complicações relacionadas ao hipercortisolismo e à hiperglicemia podem ocorrer, dificultando o controle de ambas as endocrinopatias. Este trabalho relata o caso de um gato persa diagnosticado com SC e DM. O paciente apresentava poliúria, polidipsia, seborreia seca crônica na região dorsal e nos flancos, e ausência de crescimento de pelos no abdômen. Por meio do teste de supressão com dexametasona (0,1 mg/kg) e da presença de hiperglicemia com níveis elevados de frutosamina e glicosúria, foram estabelecidos os diagnósticos de SC e DM. Foi iniciado tratamento com trilostano (2 mg/kg VO BID) e insulina glargina (2 UI/animal SC BID), resultando em estabilização da glicemia, das concentrações de cortisol e do quadro clínico do paciente, embora a fragilidade cutânea tenha persistido por um período prolongado. A investigação diagnóstica para hipercortisolismo deve ser considerada em gatos com DM para permitir o diagnóstico precoce, reduzir subdiagnósticos e melhorar a disponibilidade de dados epidemiológicos e clínicos ​​sobre essa associação. O manejo clínico descrito pode auxiliar os veterinários na antecipação de desafios terapêuticos, como a fragilidade cutânea persistente apesar do controle do cortisol.

## Introduction

Cushing’s syndrome (CS), also known as hyperadrenocorticism, is an uncommon endocrine disorder in cats (Bugbee et al., 2023). Similar to dogs, most affected cats (≈80%) present with a pituitary tumor, leading to ACTH-dependent CS (Griffin, 2021; Rapastella et al., 2023). Excess circulating glucocorticoids cause clinical signs and may lead to complications, including skin atrophy and diabetes mellitus (DM) (Bugbee et al., 2023).

DM is one of the most prevalent endocrine disorders in cats, with type 2 DM being the most common form, primarily associated with difficulties in insulin secretion, insulin sensitivity or both (Taylor et al., 2025). In dogs, Behrend et al. (2018) report that hypercortisolism is a primary cause of DM due to glucocorticoid-induced insulin resistance. Similarly, in cats, clinical manifestations are largely the result of persistent hyperglycemia (Xenoulis & Fracassi, 2022).

Fewer than 100 cats with concurrent CS and DM had been reported in peer-reviewed literature as of 2021 (Cook & Evans, 2021). In this context, the present work describes the epidemiological, diagnostic, and clinical management aspects of a Persian cat diagnosed with Cushing’s syndrome and diabetes mellitus.

## Case report

A 10-year-old spayed female Persian cat weighing 3.9 kg and negative for FIV/FeLV was presented to a university veterinary hospital. Physical examination and dermatological evaluation, including Wood’s lamp examination, were performed. Complementary tests included serum biochemistry [urea, creatinine, alanine aminotransferase (ALT), aspartate aminotransferase (AST), gamma-glutamyltransferase (GGT), glucose, amylase, total protein and fractions], complete blood count, total T4 levels via chemiluminescence, urinalysis, fructosamine measurement, and abdominal ultrasound (US). Glycemic monitoring was performed using a FreeStyle Libre® sensor.

Three months later, due to recurrence of clinical signs, additional diagnostic tests were performed, including complete blood count, serum biochemistry, abdominal ultrasound, and a dexamethasone suppression test. For this test, three blood samples were collected for cortisol measurement: at baseline (time zero), four hours after dexamethasone administration (0.1 mg/kg IV), and eight hours after administration. After diagnosis, trilostane (Vetoryl®) was prescribed at 2 mg/kg PO BID based on the attending clinician's judgment. Follow-up evaluations included clinical reassessment, fasting blood glucose, cortisol measurement, complete blood count, serum biochemistry, and abdominal ultrasound.

**Figure 1:**
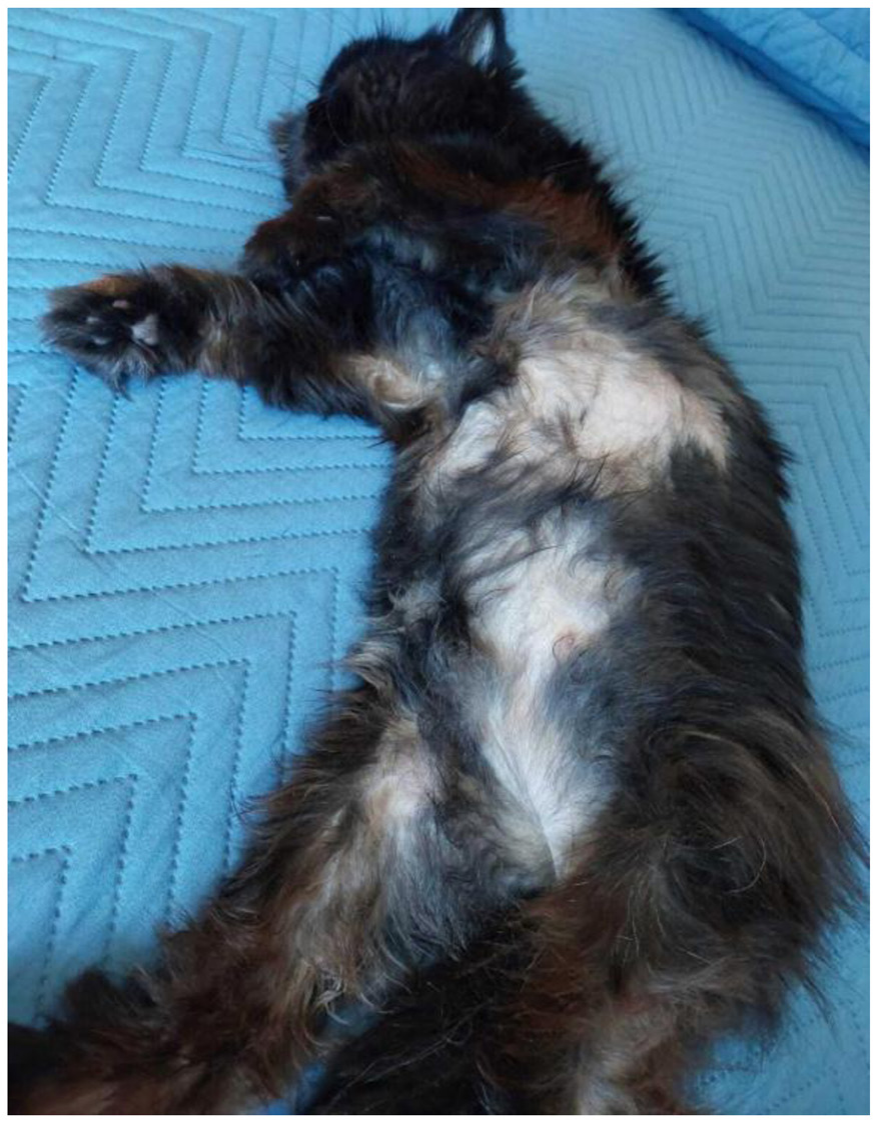
A 10-year-old Persian cat in right lateral recumbency, diagnosed with diabetes mellitus and Cushing's syndrome, presenting with alopecia in the ventral abdominal region. Source: Authors’ personal archive.

The patient exhibited polyuria, polydipsia, chronic dry seborrhea on the dorsal region and flanks, and absence of hair regrowth on the abdomen, an area that had been clipped for an ultrasound examination conducted months earlier (Figure 1). There was no pruritus, and Wood's lamp examination yielded negative results. No abnormalities were noted on physical examination.

Hyperglycemia (208.6 mg/dL; ref: 73-134 mg/dL) and glucosuria (2+; ref: negative) were observed. Fructosamine levels were above the reference range (481 μmol/L; ref: 175-400 μmol/L), confirming diabetes mellitus. Initially, glargine insulin (Lantus®) was prescribed at a dose of 0.85 IU subcutaneously every 12 hours (BID), with later adjustment to 2 IU BID after periodic evaluations (Table 1). A diabetic-specific diet (Royal Canin® Diabetic Feline) was also prescribed.

**Table 1:** Results of two glycemic curves obtained using the FreeStyle Libre® sensor in a 10-year-old Persian cat with diabetes mellitus and Cushing's syndrome, before (0.85 IU) and after (2 IU) insulin dose adjustment.

**Time of Evaluation**	**0.85 IU (Lantus^®^)**	**2 IU (Lantus^®^)**
06:00	279	197
08:00	213	180
10:00	280	136
12:00	288	111
14:00	275	184
16:00	264	153
18:00	290	158

Recommended glycemic targets for cats receiving insulin therapy: nadir glucose concentration 80-100 mg/dL and peak glucose concentration 250-350 mg/dL (Fleeman & Gilor, 2023).

**Figure 2:**
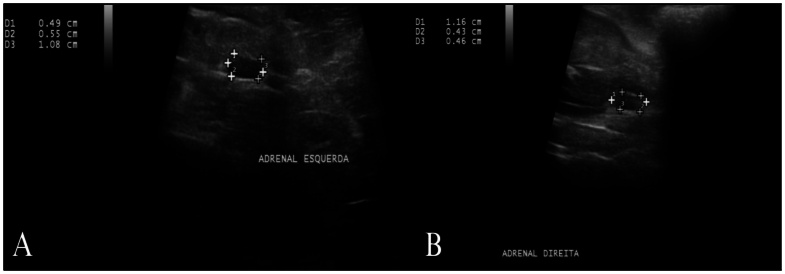
Ultrasonographic images of the left (A) and right (B) adrenal glands of a 10-year-old Persian cat diagnosed with diabetes mellitus and Cushing's syndrome, showing bilateral adrenomegaly. Source: Authors’ personal archive.

Three months later, the patient returned weighing 3.45 kg (previously 3.9 kg), presenting with feline acne and hair loss in the ventral neck and chest regions. Alopecia was also observed on the lateral thorax, where the FreeStyle Libre® sensor had been previously applied. Polyuria and polydipsia were reported again. Abdominal ultrasound revealed bilateral adrenomegaly (Figure 2) and mild hepatomegaly. Cortisol concentrations in all samples exceeded the reference range, confirming Cushing’s syndrome (Table 2).

**Table 2:** Results of the dexamethasone suppression test measured by radioimmunoassay in a 10-year-old Persian cat diagnosed with diabetes mellitus and Cushing’s syndrome.

**Parameter Assessed**	**Results**	**Reference Range** ^*^
Baseline cortisol	5.83 µg/dL	0.8-5.0 µg/dL
Cortisol 4h post-suppression	5.89 µg/dL	< 0.8 µg/dL
Cortisol 8h post-suppression	2.86 µg/dL	< 0.8 µg/dL

* Reference intervals provided by TECSA Veterinary Diagnostic Laboratory (Belo Horizonte, Minas Gerais, Brazil).

**Figure 3:**
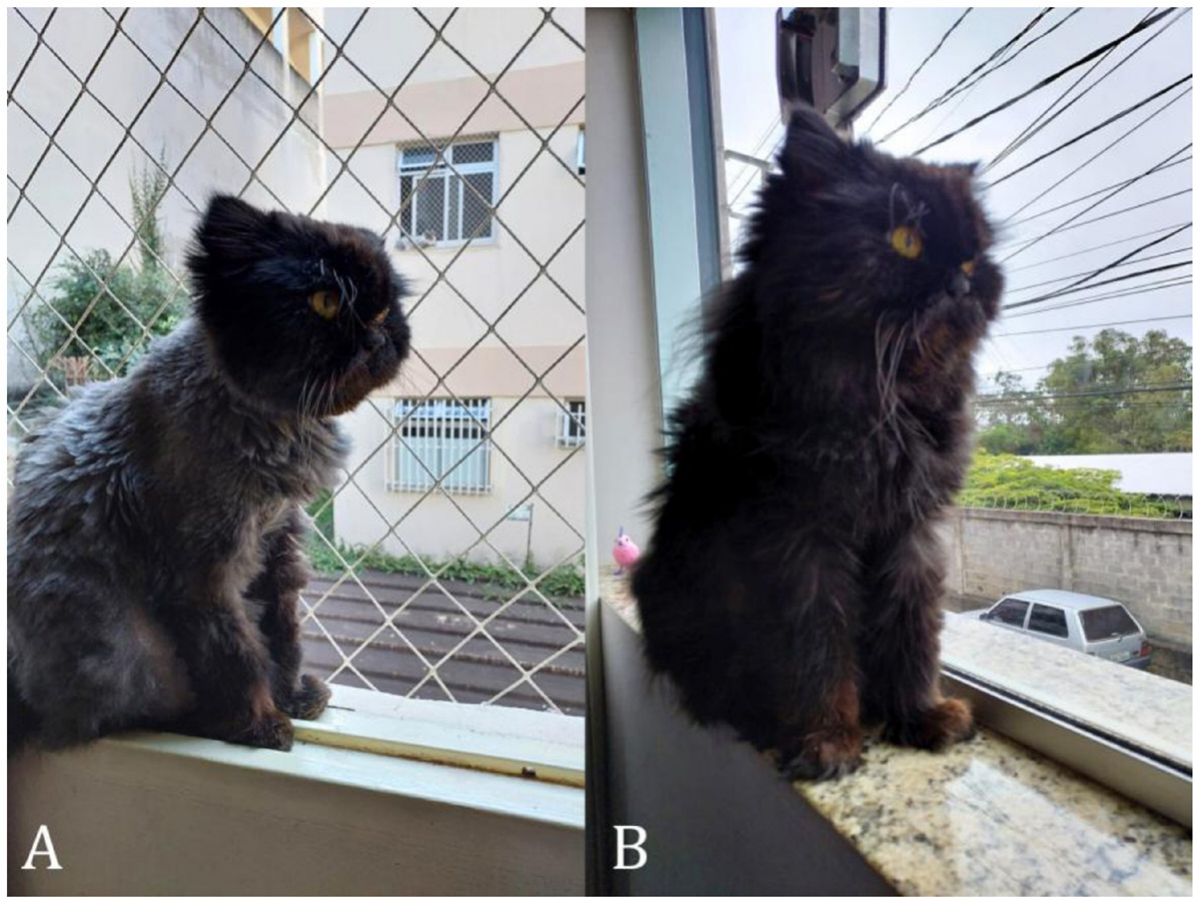
Comparison of hair growth in a 10-year-old Persian cat diagnosed with Cushing's syndrome and diabetes mellitus, before (A) and after (B) treatment with trilostane. Source: Authors’ personal archive.

Based on these findings, trilostane therapy was initiated. The patient returned 21 days later, showing stable body weight, normal urine output, thirst, appetite, and hair regrowth on the left flank. At subsequent follow-ups, hair regrowth was observed across the entire body (Figure 3). Baseline cortisol levels after trilostane treatment were 5.14 µg/dL (reference range 0.8-5.0 µg/dL), and fasting blood glucose was 130 mg/dL.

Seven months later, the owners reported recent episodes of vomiting and nausea. The patient weighed 4.0 kg at this time. Physical examination revealed a capillary refill time of 2 seconds and slightly dry mucous membranes. Complete blood count, serum biochemistry, and abdominal ultrasound showed no significant abnormalities. Due to mild dehydration (<5%), subcutaneous lactated Ringer’s solution was administered, approximately 100 mL in the interscapular region.

**Figure 4:**
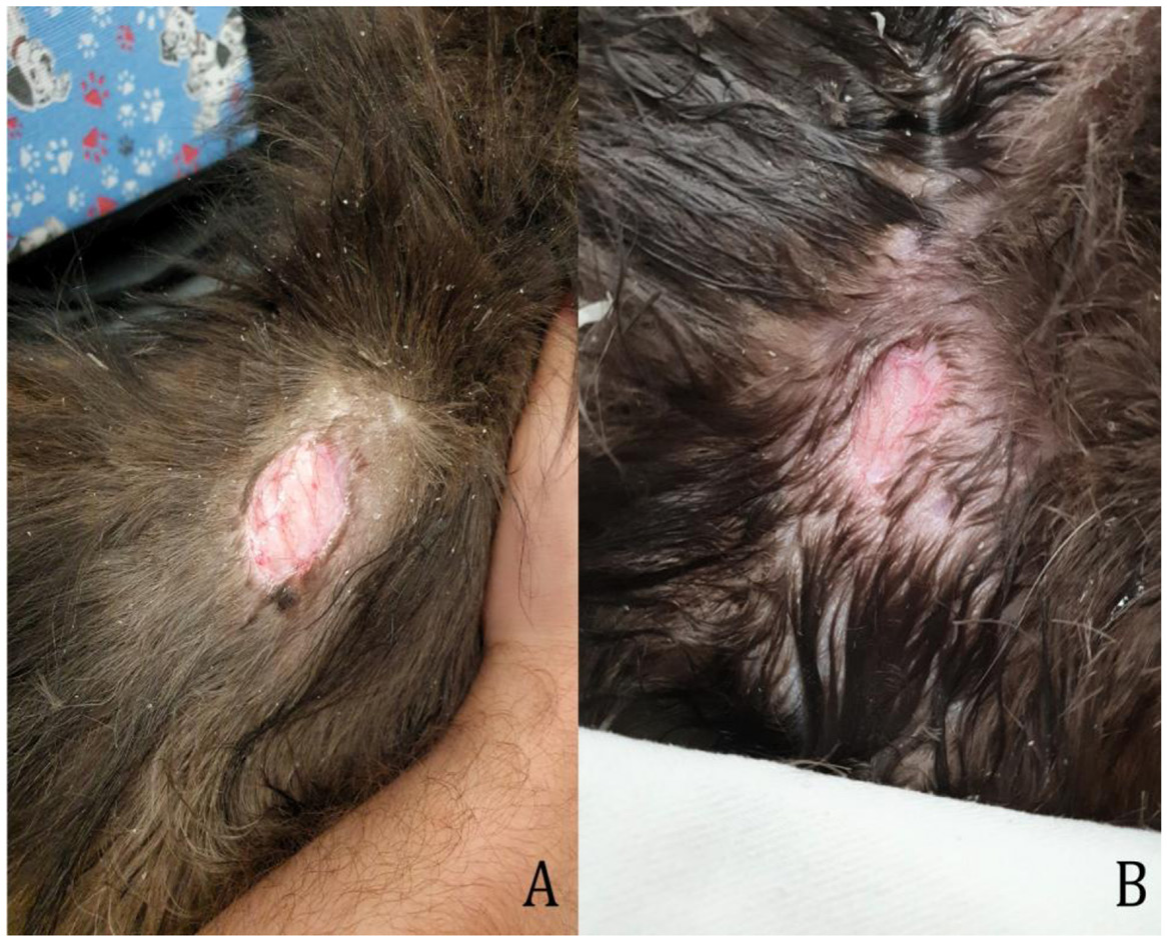
Skin lacerations near the right scapula (A) and in the right infra-scapular region (B) of a 10-year-old Persian cat with Cushing's syndrome and diabetes mellitus, following subcutaneous fluid therapy in the interscapular region. Source: Authors’ personal archive.

A few hours later, the owners noticed a circular laceration, approximately 3 cm in diameter, near the right scapula and another laceration, about 1 cm in diameter, in the right infrascapular region (Figure 4). The wound edges were approximated using cyanoacrylate glue. The skin remained stable in the following days.

## Discussion

By describing the case of a Persian cat diagnosed with Cushing’s syndrome and diabetes mellitus, this report contributes an additional case of this association in felines, whose prevalence remains unclear due to the scarcity of reports (Cook & Evans, 2021). Furthermore, the successful diagnostic approach and therapeutic management may assist veterinarians in recognizing the coexistence of these disorders, as well as in anticipating expected therapeutic challenges, such as persistent skin fragility and cortisol control difficulties.

Studies indicate that Cushing’s syndrome typically affects middle-aged cats, with an average age of 10 years (Griffin, 2021). The patient in this report was 10 years old, consistent with previously published data. The diagnosis of CS three months after DM is also consistent with the delayed diagnosis described in diabetic cats. This delay highlights the need for screening tests for CS in cats with DM using complementary methods, such as abdominal ultrasonography, the urinary cortisol-to-creatinine ratio, and serum cortisol measurement.

The literature mentions that patients with alopecia associated with endocrinopathies may not experience hair regrowth after shaving, which is why this practice should be avoided during imaging exams (Griffin, 2021). This aligns with the persistent alopecia observed in the patient after trichotomies, reinforcing the importance of investigating endocrine diseases in animals with absent hair regrowth.

More than 50% of cats with CS reportedly exhibit skin fragility due to chronic cortisol excess, which inhibits collagen synthesis (Hardy et al., 2023), rendering the skin more susceptible to lacerations. Although no specific timeline for resolution is established, skin fragility is among the clinical signs that take the longest to disappear (Hardy et al., 2023). This explains the occurrence of skin lacerations in the patient even after more than one year of appropriate treatment, despite stabilization of serum cortisol levels adequately controlled.

Although the dexamethasone suppression test is considered the definitive method for the diagnosis of Cushing’s syndrome (CS), ultrasonography (US) proved to be an effective tool for patient screening and guiding the diagnostic investigation. US is also useful for differentiating between pituitary-dependent hyperadrenocorticism (PDH) and adrenal-dependent hyperadrenocorticism, with a sensitivity of 93% (Pérez-López et al., 2021). Bilateral adrenal enlargement is the most frequently observed finding in patients with PDH (Griffin, 2021). For cats weighing less than 4 kg, adrenal gland sizes are expected to range from 2.4 to 3.9 mm (Pérez-López et al., 2021). The patient in this report had a left adrenal gland measuring 4.9 mm at the caudal pole and 5.5 mm at the cranial pole, while the right adrenal gland measured 4.3 mm at the caudal pole and 4.6 mm at the cranial pole, respectively. These findings increased the diagnostic suspicion of CS.

The low-dose dexamethasone suppression test was subsequently performed. However, in cats, a dose 10-fold higher than that used in canine protocols is required (Hardy et al., 2023). Accordingly, as recommended by Bugbee et al. (2023) and Hardy et al. (2023), a dose of 0.1 mg/kg IV was administered. The absence of cortisol suppression at 4 and 8 hours post-dexamethasone administration confirmed CS (Bugbee et al., 2023).

In this report, trilostane was chosen as the pharmacological treatment. Trilostane reduces circulating cortisol concentrations by inhibiting 3-beta-hydroxysteroid dehydrogenase, an enzyme responsible for cortisol synthesis, thereby improving clinical signs (Yayoshi et al., 2022). The recommended intervention begins with doses starting at 1 mg/kg PO, administered two or three times daily (Bugbee et al., 2023). According to Cook and Evans (2021), initial trilostane doses of up to 10 mg per cat have been reported as acceptable. Therefore, the initial dose administered in the present case was consistent with previously published recommendations.

Long-acting insulins are currently the most recommended for cats (Xenoulis & Fracassi, 2022), with glargine (Lantus®) being one of the preferred choices. To achieve glycemic control, an average dose of 2 IU per cat every 12 hours is recommended (Fleeman & Gilor, 2023), as prescribed in this report. Portable glucose monitors or sensor-based systems, such as the FreeStyle Libre^®^ used in this case, can be employed for this purpose (Xenoulis & Fracassi, 2022). According to Re et al. (2023), these devices offer ease of use and reduced stress for both caregivers and cats, allowing better glucose monitoring.

According to Fleeman and Gilor (2023), serum glucose concentrations in cats receiving long-acting insulin should remain between 80-100 mg/dL (lower limit) and 250-350 mg/dL (upper limit). The patient consistently remained within these therapeutic targets after insulin dose adjustment.

Studies indicate that cats receiving appropriate insulin doses twice daily, combined with a low-carbohydrate diet, have a better prognosis, including the possibility of remission (Gottlieb et al., 2024). Therefore, in addition to insulin therapy, diabetic patients should receive a high-protein, low-carbohydrate diet (Benedict et al., 2022). Accordingly, the patient was maintained on therapeutic food specifically designed for diabetic cats, contributing to successful treatment.

## Conclusion

This work contributes to the literature by adding a case to the limited number of reports describing the association between Cushing’s syndrome and diabetes mellitus in cats. Given that the diagnosis of CS in cats with diabetes mellitus frequently occurs late, these patients should be screened for this endocrinopathy. Furthermore, it highlights persistent skin fragility associated with CS for several months, even after adequate control of serum cortisol concentrations. Finally, the management of both endocrine diseases was essential for the effective control of clinical signs, contributing to an improved prognosis and quality of life.
